# Unintended coronary biopsy following successful management of rotational atherectomy burr entrapment using the Ping-Pong technique: images in cardiology

**DOI:** 10.1093/ehjcr/ytaf374

**Published:** 2025-08-13

**Authors:** Dimitrios Siamkouris, Marc Schloesser, Elmar Offers, Stergios Tzikas

**Affiliations:** Department of Cardiology, Dreifaltigkeits Hospital Lippstadt, Academic Teaching Hospital of Westfaelische Wilhelms University Muenster, Klosterstrasse 31, Lippstadt 59555, Germany; Department of Cardiology, Dreifaltigkeits Hospital Lippstadt, Academic Teaching Hospital of Westfaelische Wilhelms University Muenster, Klosterstrasse 31, Lippstadt 59555, Germany; Department of Cardiology, Dreifaltigkeits Hospital Lippstadt, Academic Teaching Hospital of Westfaelische Wilhelms University Muenster, Klosterstrasse 31, Lippstadt 59555, Germany; Third Department of Cardiology, Ippokrateio General Hospital, Aristotle University of Thessaloniki, Leoforos Kostantinou Karamanli 50, 54642 Thessaloniki, Greece

## Case summary

We present the case of an 81-year-old female patient with unstable angina. Coronary angiography revealed ostial left main disease and heavily calcified proximal left anterior descending artery (LAD) stenosis. Rotational atherectomy was selected as the intervention strategy. Unexpected burr entrapment leading to LAD occlusion occurred, but was successfully managed using the Ping-Pong technique. This involved advancing a second guidewire past the burr into the distal LAD and performing subsequent balloon dilatations for plaque modification, which facilitated burr dislodgement. Notably, a significant calcified coronary plaque specimen was retrieved with the burr, equivalent to an unintentional coronary biopsy. The intervention was finalized with the implantation of three stents and the patient was discharged 2 days later. This case highlights the need for caution during and after burr retrieval due to the risk of coronary artery injury, which can range from inadvertent biopsies to severe perforations, necessitating urgent pericardiocentesis.

## Case description

An 81-year-old woman with a history of hypertension, dyslipidaemia, diabetes, and a previous minor stroke presented with unstable angina. Physical examination, ECG, and high-sensitivity troponin levels were unremarkable.

**Figure 1:**
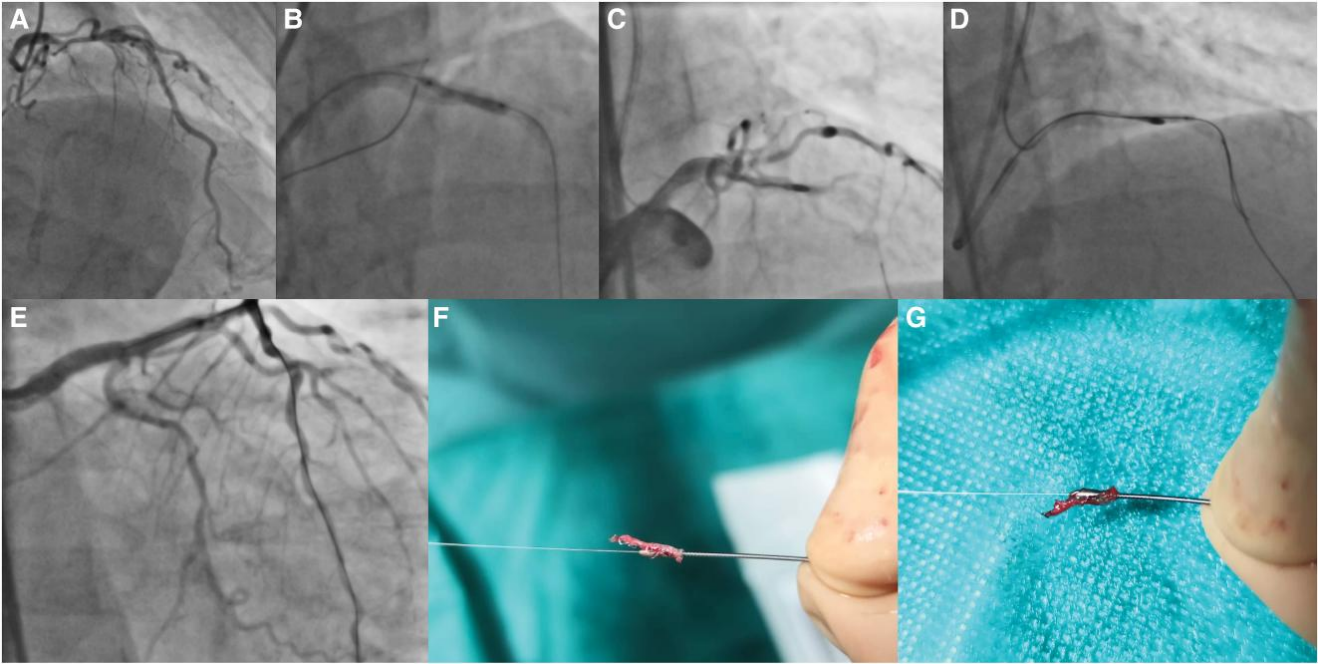
Ostial left main and calcified proximal LAD stenosis (*A*), balloon dilatation with dog bone effect due to heavy calcification (*B*), Burr entrapment (*C*), Ping-Pong technique, wiring besides burr, and finally burr dislodgement with balloon dilatations next to it (*D*), final result after implantation of 3 stents (*E*), and coronary biopsy specimen after burr retrieval (*F and G*).

Coronary angiography via radial artery revealed ostial left main disease and heavily calcified proximal LAD stenosis (Supplementary  *Video 1*, calcified stenosis) with an FFR value of 0,67 (*Figure 1A*). Primary rotational atherectomy strategy was abandoned, due to abortive advancement of the microcatheter. However, successful lesion preparation with small diameter balloons (*Figure 1B*) facilitated subsequent microcatheter placement. A Rotawire floppy was introduced and rotational atherectomy was performed with a 1,5 mm burr at 180.000 rpm, employing a pecking motion (Supplementery  *Video 2*, Rotablation). During the procedure the burr unexpectedly stalled, causing severe angina, but without hemodynamic instability. Angiography revealed acute LAD occlusion without signs of perforation (*Figure 1C*).

Burr retrieval via controlled traction was unsuccessful, prompting establishment of femoral access for a secondary guide catheter, initiating the Ping-Pong technique (Supplementary  *Video 3*, Burr Entrapment with LAD occlusion and wiring in Ping Pong Technique). A Fielder XTA wire advanced distal to the entrapped burr and sequential balloon dilatations (Supplementary  *Video 4*, Ballon dilatation next to the Burr) facilitated plaque modification and burr dislodgement (*Figure 1D*). A sizable calcified plaque specimen was found attached to the retrieved burr (*Figure 1F* and *1G*. No coronary perforation was observed.

The procedure concluded successfully with the implantation of three stents extending proximally to the left main ostium in cross over technique, followed by flaring and proximal optimization (Supplementary  *Video 5*, final result) (*Figure 1E*). The patient was discharged in stable condition 2 days post-procedure and maintained clinical improvement at 3-year follow-up.

Burr entrapment is a rare but potentially severe complication with an incidence ranging from 0,4% to 0,8%,^1^ which has to be approached with prudence. A double guide catheter Ping-Pong technique could be considered as a solid first line approach. However, caution is required after burr dislodgement. The extensive mechanical forces advanced in the coronary system to disengage the burr, could lead to major injury of the coronary arterial wall. This can manifest as an unintentional coronary biopsy, which in cases of rotational atherectomy within an unexpanded stent can even lead to partial extraction of an entangled stent.^2^ In the worst case scenario, severe coronary perforation may occur,^3^ frequently necessitating immediate pericardiocentesis.

## Lead author biography


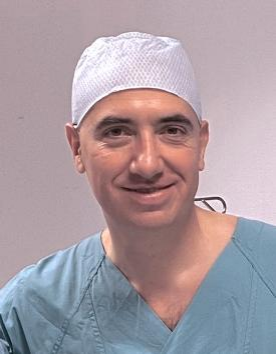


Dimitrios Siamkouris, graduated 2005 from the School of Medicine at Aristotle University of Thessaloniki in Greece. He completed his cardiology residency in 2013 in Dreifaltigkeits Hospital Lippstadt and in University heart center Hamburg in Germany, where he specialized in terminal Heart Failure and management of heart transplant patients, earning his doctoral degree in this field. He further specialized in Coronary Intervention field and Cardiac Device implantations receiving multiple Certifications. Since 2018 he serves as the Leading Consultant of the Cardiac Catheterization Laboratory and Cardiac Device Implantation section in Dreifaltigkeits Hospital Lippstadt, academic teaching Hospital of WestfaelischeWilhelms University Muenster.

## Supplementary Material

ytaf374_Supplementary_Data

## Data Availability

All data are incorporated into the article.
